# Interference of *Lactobacillus casei *with *Pseudomonas aeruginosa *in the treatment of infected burns in Wistar rats

**DOI:** 10.22038/IJBMS.2020.47447.10920

**Published:** 2021-02

**Authors:** Mohammad Abootaleb, Narjes Mohammadi Bandari, Nazila Arbab Soleimani

**Affiliations:** 1 Department of Biology, Qom Branch, Islamic Azad University, Qom, Iran; 2 Department of Microbiology, Damghan Branch, Islamic Azad University, Damghan, Iran

**Keywords:** Biofilms, Multidrug-resistant, Probiotics, Pseudomonas infections, Wound healing

## Abstract

**Objective(s):**

Burns are the most common type of trauma with a high mortality rate worldwide. The use of modern and natural medicines, especially probiotic products, has been recently considered for cutaneous wound healing. The present study was designed to investigate the effect of *Lactobacillus casei *on wound healing caused by *Pseudomonas aeruginosa*.

**Materials and Methods:**

In this study, the anti-adhesion activity of* L. casei* was examined by the glass slide method, and inhibitory substances in the cell-free supernatant (CFS) were quantified by high-performance liquid chromatography (HPLC). Following the induction of second-degree wounds, multidrug-resistant (MDR) *P. aeruginosa* was injected subcutaneously and directly on the burn. The animals were divided into four groups. The supernatant of *L. casei *was sprayed for treatment every day and wound healing was examined.

**Results:**

Based on our findings, the supernatant of* L. casei* showed considerable anti-adhesion effects on *P. aeruginosa*. HPLC analysis indicated that the inhibitory effect of this supernatant can be due to four main organic acids including lactic acid, acetic acid, citric acid, and succinic acid. The effect of treatment on fibroblastic cells showed that the treated group by supernatant of* L. casei* had more fibroblastic cells compared with the non-treated group. Moreover, this supernatant increased the rate of fibroblastic cells, re-epithelialization in the wound area, and the largest thickness of the epidermis and dermis layers.

**Conclusion:**

The present findings showed that* L. casei* supernatant significantly reduced inflammation and could be used to treat *P. aeruginosa *infection in second-degree burns.

## Introduction

Tissue damage can result from physical, chemical, and biological burns. Depending on the type and duration of tissue repair, it can be divided into chronic and acute types. Burn wounds are recognized as a type of tissue damage (1, 2). They are usually classified into three degrees. The epidermal and dermal layers of the skin are involved in second-degree burns. These burns are commonly accompanied by blisters and fluid buildup-related abdominal pain and are also extremely sensitive to touch (3, 4).

Severe ulcers, which can be categorized as burn wounds, require an average of three weeks to heal. Due to the high prevalence of burns in both developing and developed countries, burn trauma has been recently taken into consideration. The trauma associated with burns, especially when excision and grafting of burn eschar are contraindicated, may lead to various complications, including wound infection. On the other hand, wound healing has been always a health concern. Several medications and ointments, such as silver sulfadiazine, have been used for burn wounds, although they have several limitations and side effects (5, 6).

*Pseudomonas aeruginosa* is a Gram-negative bacterium and one of the greatest threats to patients with severe partial- and full-thickness burn injuries. It is the most important human pathogen in the *Pseudomonadaceae* family and the second leading cause of burn infections. The extreme use of antibiotics has increased the antibiotic resistance of *Pseudomonas *species worldwide. Multidrug-resistant (MDR) *P. aeruginosa* infection is a serious problem in treating hospitalized patients with burns (7- 9). 

Most pathogenic bacteria must attach to the target cell surface, leading to biofilm formation in various environments. Biofilm formation is one of the causes of antibiotic resistance and failure to treat bacterial infections (10- 12).

Rapid specialized treatment is usually necessary for patients with severe burn injuries. In today’s world, biotechnological advances have focused on the use of natural metabolites that inhibit the growth of pathogenic microorganisms and can be desirable alternatives to chemical preservatives. A major group of inhibitory compounds is produced by probiotic bacteria. Probiotic microorganisms have beneficial effects for consumers, as they affect their health through multiple mechanisms by the production of antimicrobial components. Also, probiotic bacteria have anti-inflammatory and regenerative activities in the treatment of skin traumas (13, 14). 

Animal models of burn wounds are essential for the evaluation and improvement of therapeutics for burn wounds. Several animal models, including mouse models, are extensively used to study the burn wound healing process. Burns can be mimicked in experimental animal models to evaluate the patterns of burn wound healing. The selection of an appropriate method to induce burns, besides the use of constant water temperature to cause the same burn in all specimens, is crucial (15, 16).

This study aimed to evaluate the antimicrobial and macroscopic effects of *L. casei *supernatant as a topical treatment for burn wounds associated with *P. aeruginosa* infection. 

## Materials and Methods

***Microorganisms***

*Pathogenesis bacteria*

The *P. aeruginosa* colony was identified from hospitalized burn patients in Firoozgar Hospital of Tehran via Gram staining, as well as standard biochemical tests, including oxidase test, catalase test, Indol test, Methyl Red and Voges Proskauer (MR-VP) test, motility test, citrate test, and fermentation of sugar types (TSI) and color production were done (17, 18).

*Probiotics bacteria*

*L.* *casei* PTCC 1608 was the probiotic bacteria used in the experiments prepared from the Persian Type Culture Collection of the Iranian Research Organization for Science and Technology. The bacterium was cultured anaerobically for one day at 37 °C in the liquid MRS medium. The CFS was prepared by centrifugation of an overnight culture of probiotic bacteria filtered with a 0.2 μm pore size filter (8, 19).

***Biofilm formation***

*Glass slide method*

To investigate *P. aeruginosa* adhesion on the glass slide, the overnight culture of* P. aeruginosa* (1 ml; 10^8^ CFU/ml) was inoculated into a flask, containing 100 ml of sterile brain-heart infusion (BHI) broth, supplemented with 1% sucrose. The glass slides were washed in distilled water twice after rinsing the detergent solution. Next, they were dried, transferred to flasks, and autoclaved at 121 °C for 15 min. The flasks were then transferred to a rotational incubator (100 rpm) at 35 °C for 18–20 hr. Following that, the glass slides were removed from the flasks and rinsed twice with 10 ml of PBS solution to eliminate unattached cells. The glass slides were finally stained with 2% crystal violet for 5 min, rinsed, exposed to air, and visualized under an optical microscope, equipped with a digital camera (20).

*Scanning electron microscopy (SEM)*

First, the samples were fixed with glutaraldehyde in cacodylate buffer, and then, exposed to different ethanol dehydration series. Afterward, they were subjected to a dehydration concentration of 100% ethanol plus hexamethyldisilazane 2×100% (HMDS, Ted Pella, USA). All coupons were covered with a thin palladium-gold layer. Finally, coaggregation was observed using an SEM system (FEI Quanta 400 FEG ESEM/EDAX Genesis X4M, FEI Company, USA) in a high-vacuum mode at 15 kV (21).

***Anti-adhesion activity***

The effect of *lactobacilli* CSF on the* P. aeruginosa* adherence was determined with a glass slide method. To investigate *P. aeruginosa* adhesion on the glass slide, 1 ml of overnight cultured *P. aeruginosa* (10^8^ CFU/ml) was inoculated into a flask containing 100 ml of sterile BHI broth supplemented with 1% sucrose and one slide with and without 10 ml *L. casei* supernatant. The glass slides were washed in distilled water twice after cleaning detergent solution, then dried and transferred to flasks then autoclaved at 121 °C for 15 min. After that, the flasks were transferred to a rotational incubator (100 rpm) at 35 °C for 18–20 hr. Then, the glass slides were taken off from the flasks and rinsed twice with 10 ml of PBS solution to eliminate unattached cells. The glass slides were stained with 2% crystal violet for 5 min, rinsed, exposed to the air, and a snapshot was taken by an optical microscope with a digital camera (22).

***High-performance liquid chromatography (HPLC) analysis***

Organic acids were quantified by the HPLC method. *L. casei* strains were cultured in MRS broth medium for 24 hr. CFS was prepared by centrifugation of an overnight culture of probiotic bacteria and filtered with a 0.2 μm pore size filter. The filtrate was then re-cultured for 72 hr in the MRS broth medium to make sure that it is sterile. Twenty µl of the filtrate was injected into the HPLC system. Chromatographic separation was achieved by reverse phase column chromatography (C18 column) through an aqueous mobile phase. The UV absorbance was recorded at 60 °C. Quantification of probiotic supernatants was performed on an Agilent 1100 series HPLC system (23, 24).

***Animals***

In the present study, 30 healthy intact male Wistar rats were used. The experimental animals were purchased from the Pasture Institute of Tehran, Iran. The animals were randomly divided into 5 groups (n=6) and housed in a standard animal room (temperature 20–22 °C, 12-12 hr dark-light cycle, 37% humidity). They had free access to water and food pellets before and during the experiments at all times (25, 26). 

***Burn Wounds***

The study protocol was approved by the Animal Ethics Committee of Islamic Azad University, Qom branch. The rats’ backs were shaved immediately before burning, then they were anesthetized by intraperitoneal injection of ketamine. A cone-shaped metallic device, made of iron with a diameter of 1 cm was used for the creation of burn wounds. The skin was for 5 sec heated by a heated metal in boiling water. Acetaminophen oral solution was administrated to the rats for enhancing the pain thresholds (27). 

*Groups and induction of second-degree burn wounds*

After induction of the second-degree wounds, MDR *P. aeruginosa *was dropped on the burn surfaces with 200–300 CFU (day 0). The animals were divided into four groups (n=6) as follows: the negative control (no drugs were used in this group), control (silver sulfadiazine 1% treatment as a gold standard treatment), *L. casei* supernatant treatment groups. The supernatant was sprayed for treatment every day. The groups were observed for 28 days (28, 29). 

*Wound measurement area (Ruler method)*

Assessment of the wound tissue healing process was done through visual inspection and wound size measurements (width and length) through a disposable ruler at the same time per week (30- 32).

The assessment of wound healing process was applied through two formulas:

1) Open wound area (% OWA):

% OWA = (WAn) / WAo × 100

(WAn wound area size per week and WAo wound area size on day zero)

2) Wound healing rate:

Wound healing rate = (wound area size on day zero- wound area size on day X) / wound area size on day zero ×100

(X= on days 7, 14, 21, and 28)

***Histopathological study***

Histopathologic follow-up examinations were used for the skin samples taken from each group on the 7^th^, 14^th^, and 21^st^ days. The following processes were applied: The biopsy specimens were fixed in 10% formaldehyde followed by paraffin embedding. Then, the samples were stained using Hematoxylin-Eosin (H&E) staining techniques. 

In the next step, the histopathologic examination was done under an Olympus optical microscope and performed by 2 pathologists blinded to the experimental groups. Finally, inflammation, re-epithelialization, neovascularization, the occurrence of granulation tissue, and collagen accumulation were considered in these specimens via the scoring system used for histopathologic examination, as previously described (33, 34).

***Statistical analysis***

The collected data were analyzed through one-way ANOVA and Tukey tests by using SPSS software version 23.0 (SPSS, Chicago, IL). *P*-value<0.05 was considered as significant level.

## Results

***Antibiotic susceptibility and Biofilm formation***

**Figure 1:**
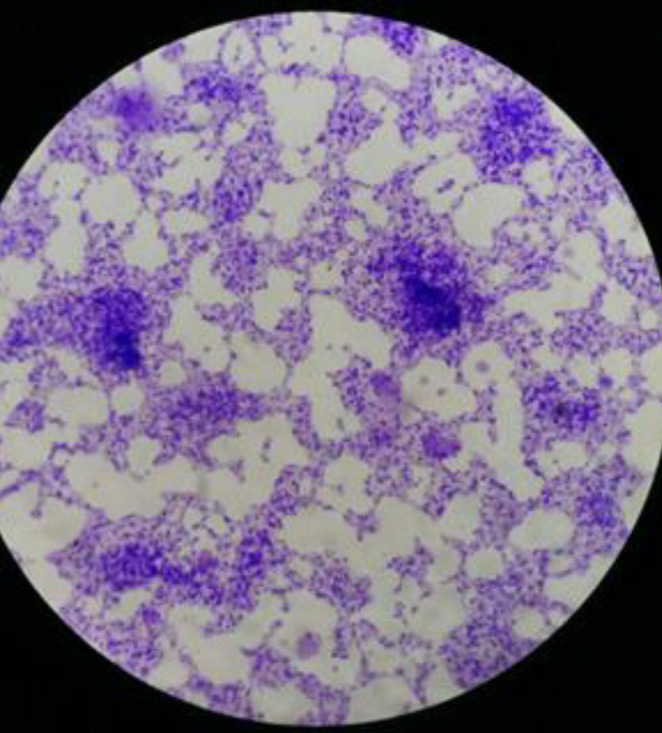
*Pseudomonas aeruginosa* biofilm formation on the glass slide

**Figure 2:**
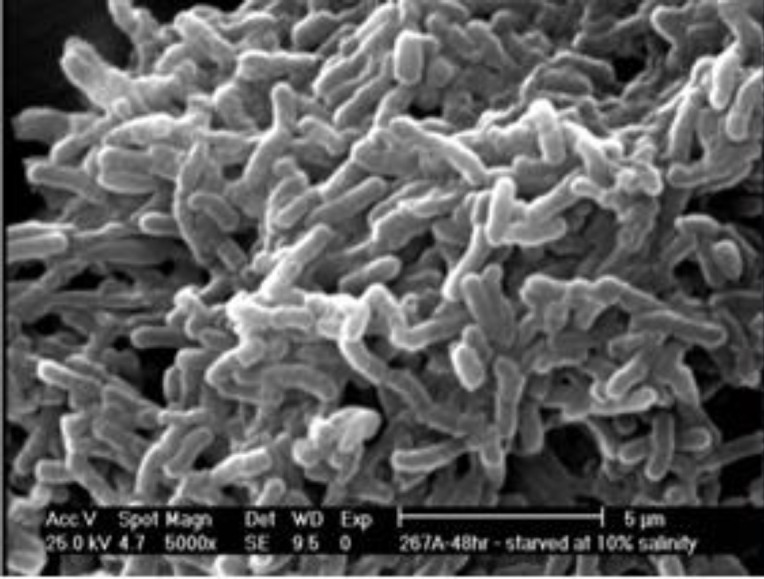
Scanning electron microscopy (SEM) of *Pseudomonas aeruginosa *biofilm cells

Out of all *P. aeruginosa* isolates one of them was selected with multi-drug resistance. After that*, P. aeruginosa* biofilm was identified using glass slides and SEM methods (Figures 1 and 2).

***Anti-adhesion activity***

**Figure 3:**
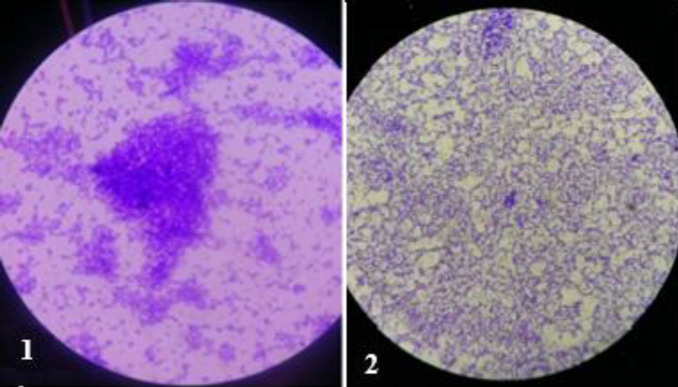
*Pseudomonas aeruginosa *adhesion on the glass slide. 1: Control group (in the absence of *Lactobacillus casei* supernatant). 2: Experimental group (in the presence of *L. casei* supernatant)

The glass slide method proved the presence of *L. casei* supernatant dramatically reduced the process of attachment (Figure 3).

***High-performance liquid chromatography (HPLC) analysis***

**Figure 4:**
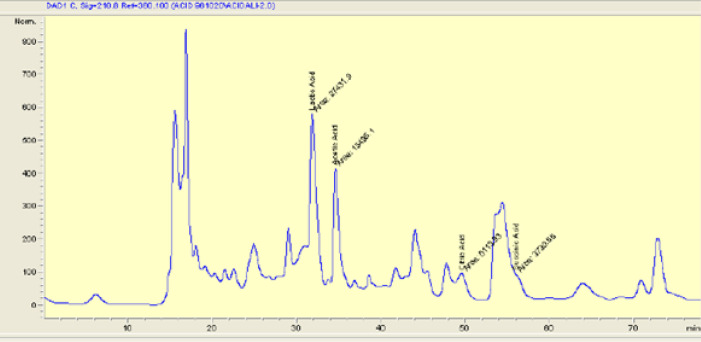
Presence of different organic acids analyzed by HPLC. LA: lactic acid, AA: acetic acid, CA: citric acid, SA: succinic acid

HPLC analysis indicated that supernatant inhibitory effect can be due to the main organic acids, including lactic acid, acetic acid, citric acid, and succinic acid. Figure 4 shows the presence of different organic acids using HPLC analysis.

***Burn wound healing***

**Figure 5:**
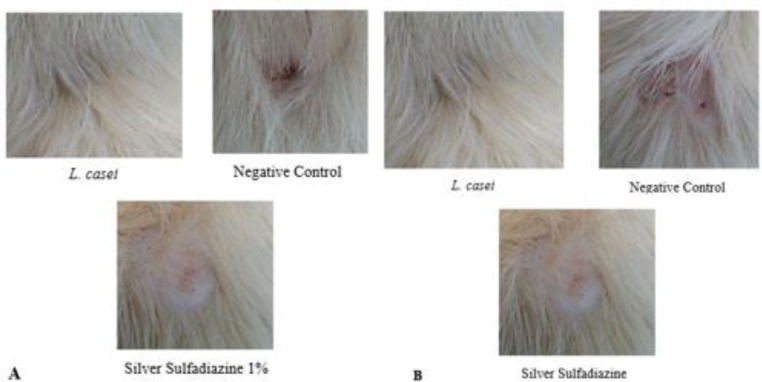
The appearance of the burn wound area in treated groups with (supernatant of L.casei and Silver sulfadiazine) at Wistar rats on; A. 21 days and B. 28 days

The effect of treatments on fibroblastic cells showed that the group treated by supernatant of *L. casei* had the most number of fibroblastic cells compared with the non-treated group. Also, the mean number of fibroblastic cells indicated that there was a significant difference in the mean number of fibroblastic cells between the group treated by the supernatant of *L. casei*, sulfadiazine, and non-treated group on day 28 (Figure 5). The results from the assessment of the weekly wound surface area are shown in Table 1.

***Histopathological study***

**Figure 6:**
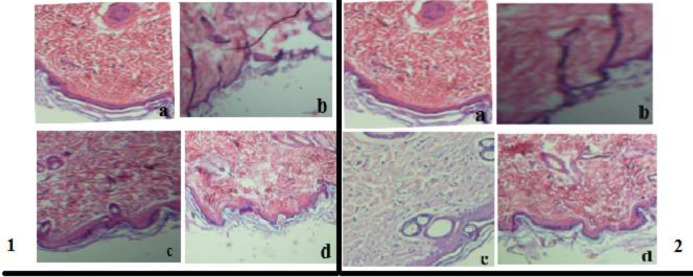
1. H&E staining showing the histological changes of mouse skin in the unburned and burned mice; 6. 1. H&E appearance of unburned and burned mouse skin on the 21^st^ day; (a. Normal skin b. Negative control group c. Group treated by the supernatant of *Lactobacillus casei* d. Group treated by silver sulfadiazine), 6. 2. H&E appearance of unburned and burned mouse skin on the 28^th^ day; (a. Normal skin b. Negative control group c. Group treated by the supernatant of* Lactobacillus casei *d. Group treated by silver sulfadiazine)

The results of the burn wound healing process by sectioning and H&E staining on the burn day 28 showed the largest thickness of the epidermis and dermis layers in the treated group with *L. casei* compared with another group (silver sulfadiazine) indicating the high efficiency of the supernatant *L. casei* (*P*<0.05) (Figure 6). 

**Table 1:** Area measurement via ruler based method

28	21	14	7	days Test results	Treatment
**4**	9	49	100	Wound measurement area percentage	**Control**
**96**	91	51	0	Wound healing rate percentage	
**0**	0	9	25	Wound measurement area percentage	***Lactobacillus casei***
**100**	100	91	75	Wound healing rate percentage	
**0**	4	25	100	Wound measurement area percentage	***Silver sulfadiazine***
**100**	96	75	0	Wound healing rate percentage	

## Discussion

Bacterial adhesion to host cells is one of the essential steps in the development of infections (34, 35). It can be classified into two major categories, that is, non-specific and specific (35, 36). Non-specific adhesion is reversible and occurs by physiochemical forces. In this type of adhesion, mobile bacteria move toward chemoattractants, such as glucose and oligopeptides, and grow on surfaces. Chemotaxis regulates and prepares bacteria-host cell interactions (35, 36). On the other hand, specific adhesions are irreversible and occur when specific bacterial adhesions (e.g., capsules, pili, and some exopolysaccharides) interact with host cell receptors, which contribute to initial bacterial infections. Today, researchers believe that by preventing bacterial-host cell interactions, infections can be avoided (37- 39).

Due to scarcity of data on the consumption of antibiotics, the prevalence of MDR bacteria is on the increase, which may be threatening for human health; therefore, researchers are seeking new safe alternative treatments (40). According to many studies, probiotic bacteria, especially *lactobacilli* and their secondary metabolic products, can be proper candidates for controlling and preventing infections (41, 42). In the present study, the antimicrobial and anti-adhesive effect of *L. casei* supernatant against *P. aeruginosa *in burn wounds was investigated.

There are various analytical techniques for the measurement of organic acids, such as titrimetric methods, gas chromatography, colorimetric assays, and enzymatic methods (43). The HPLC method is a separation method for the quantitative measurement of organic acids within a short period, which requires the minimum sample volume for separating one or more organic acids (44). In the present study, the HPLC analysis showed that the supernatant of *L. casei* contained high concentrations of lactic acid, acetic acid, citric acid, and succinic acid. These results showed that the increase in organic acids, especially lactic acid, may account for the remarkable antagonistic activity of *L. casei*.

In the current study, the antagonistic effects of probiotic *Lactobacilli *against *P. aeruginosa *were evaluated. The result showed that the *L. casei* supernatant had a good inhibitory effect on *P. aeruginosa *growth. Vuotto *et al.* (2014) reported that probiotic culture and their supernatant were able to inhibit *P. aeruginosa *burn infections (45). One of the main properties of probiotic *Lactobacilli *which makes them so potent to antagonize pathogenic bacteria is the production of antimicrobial substances (46, 47). Some studies have demonstrated that the antibacterial compounds of probiotic *Lactobacilli* can interact with the bacterial cell membrane by reducing the pH of the environment, denaturing proteins, and finally, eradicating pathogens through the physiology and morphology of the cytoplasmic membrane of bacteria and leakage of its content (42).

Today, several distinct surface *Lactobacilli* proteins are predicted to enhance binding to the surface of pathogenic bacteria, which inhibits pathogen attachment and colonization (48). As described in other studies, probiotic bacteria through coaggregation with pathogenic bacteria can prevent their growth and attachment to host cells, as confirmed in the present study (47). Since the anti-adhesive effects of probiotic bacteria are vital for preventing the primary stage of infection, the anti-adhesive activity of *L. casei* was investigated in the current study. Researchers have highlighted the anti-adhesive role of probiotic bacteria, but the mechanisms of their action remain undetermined (45, 48). A *study* (2019) demonstrated that* P. aeruginosa *colonization and adhesion were inhibited by *Lactobacillus plantarum* supernatant (49). Also, Bienenstock *et al.* (2013) demonstrated that probiotic bacteria prevent pathogenic bacteria attachment due to competition for specific host cell receptors (50).

In the current study, macroscopic and microscopic assessments of the wound area, treated with the supernatant of *L. casei*, indicated a greater recovery than the control group, especially on days 21 and 28 after burning induction. The pathological findings revealed that *L. casei* significantly accelerated the repair process in full-thickness wounds on day 21. Our experimental results are consistent with the findings of some previous studies, in which the positive role of topical application of probiotic bacteria in the wound healing process was documented. 

Researchers (2017) evaluated the effect of local treatment of *second-degree* *burns *with *L. acidophilus* on male rats. Their results showed that the treatment of burn wounds with *L. acidophilus* could accelerate the recovery of second-degree burn wounds (27). Besides, they found that probiotic bacteria exerted beneficial effects on different aspects of the wound healing process, such as the reduction of the inflammatory response and acceleration of granulation tissue formation and re-epithelialization.

According to one study (2012), the use of kefir grains for the treatment of thermal burns led to the faster recovery of ulcers, compared with treatment with silver sulfadiazine (28). 

## Conclusionn

With the increasing incidence of MDR *P. aeruginosa* in hospitals and clinics, new treatments are necessary for preventing the pathogen’s growth without inducing greater resistance. These results indicated that the supernatant can be successfully and conveniently used for treating the mouse model of burn wounds. They are easy to use, cost-effective, and safe for use by humans and they also can be used as alternatives to chemical drugs.
